# Learning Disabilities and Medical Students

**DOI:** 10.15694/mep.2018.0000142.1

**Published:** 2018-07-11

**Authors:** Arslaan Javaeed

**Affiliations:** 1 University of Ottawa

**Keywords:** Learning disability, awareness, challenges, medical students

## Abstract

This article was migrated. The article was marked as recommended.

**Background:** Learning disabilities (LD) are a mixed group of disorders exhibited by substantial difficulties in the achievement and use of listening, speaking, reading, writing, reasoning, or mathematical skills.

**Objective:** The objective of this paper is to highlight different learning disabilities and their effects on medical students and suggest the best assessment strategy for such students.

**Methods:** The medical education literature was searched for articles related to learning disabilities and how they effect medical students.

**Results and Conclusion:** Learning disability constitutes to be challenging for the student as well as the faculty because apparently, there is no disability to be seen. It is difficult to diagnose and sometimes it remains unidentified till late adulthood when the compensatory mechanisms crash down leaving the student in despair and low self-confidence. The identification of such a disorder requires appropriate personnel to diagnose such condition which are currently not available in the medical schools. Literature shows that multiple choice questions (MCQs) are the best method of choice in assessment of students with learning disability as it does not discriminate between students with and without LD.

## Introduction

Learning disabilities (LD) are a heterogenous group of disorders with a prevalence of almost 6% in students entering higher education ( Ricketts, Brice and Coombes, 2010). The disability is usually not known until students cannot cope with the increasing burden of studies. A study revealed that almost 66% of the students were not aware of their learning disability when they entered college ( Rosebraugh, 2000). The compensatory mechanisms evolved for the disability, throughout the students’ early education, prove to be insufficient in dealing with medical education which is one of the toughest undergraduate courses. This leads to their poor performance and lack of self-confidence. The acceptance and disclosure of the learning disability is itself a stigmatizing process due to general lack of awareness of public and academia. Early recognition of these learning disabilities and provision of proper accommodation can lead to dramatic changes in the learning process of these students. In this paper, I will be discussing a brief description of the learning disabilities followed by two dominant themes. Firstly, the attitude and disclosure of LDs by medical students and how we can bring a change in the attitude of medical students. Secondly, the instructional and assessment approach for these students along with the teaching methods required for medical students about LD and their assessment. At the end, I would like to discuss the prevailing situation of recognition of this disorder in my medical college with a view to offer suggestions to improve it.

## Learning Disabilities

According to *Diagnostic and Statistical Manual of Mental Disorders* (DSM- 5), Specific learning disorder (SLD) is a “neurodevelopmental disorder that begins by school age, although it may be recognized until later. It involves ongoing problems learning key academic skills, including reading, writing and math. These are roughly divided into specific and non-specific groups ( Ali *et al.*, 2017).

1. Specific LDs:

i. Dyslexia
ii. Dyscalculia
iii. Development coordination disorder (DCC)
iv. Attention deficit disorder (ADD)
v. Attention deficit hyperactivity disorder (ADHD)

2. Non-Specific LDs:

It is attributed to difficulty in or any combination of:

i. Auditory processing
ii. Visual processing
iii. Speed of processing

Another classification proposed by Accardo et al divides learners into two categories: *sequential learners*, analytical learners who follow a logical pattern, and *simultaneous learners,* those who have a comprehensive approach to a subject and understand the concept instead of specific steps ( Rosebraugh, 2000). Students having LDs usually try to adapt by over-working one system in compensation to the deficient one.

LDs are alleged to be caused by dysfunction of central nervous system, inherent to the individuals and are permanent. The disability is not the result of specific instructional method, organic medical disorder, difficulty in language comprehension or socio-economic circumstances. The criteria for diagnosing depends on a number of observations such as individual’s family and medical history, academic results, history of having difficulty in learning, educational tests, standardized tests and specific psychological tests, and interviews. Once diagnosed, the severity of disorder can be evaluated, and appropriate provisions made to encourage further learning. The medical community appears to be very slow in acknowledging LDs. Nevertheless, efforts are being made to aware people about these disabilities and enhance their recognition. One such example is an article in New York Times which describe LDs as difficulty learning to read despite sufficient intelligence ( Rosebraugh, 2000).

## Learning disability in Medical Students

Half of the individuals with LDs are not identified before they start their college. Alarmingly, another study revealed that freshmen reporting LDs increased from 15.3 to 32.3% from 1988 and 1994 ( Rosebraugh, 2000). A study also showed that LDs among medical students rose from 1.34 to 2.90 per school varying from 0-16 students per school ( Rosebraugh, 2000). Another study reports a total of 3% suffering from LD which were not known before. This alarming prevalence of LD in medical schools point to the fact that a lot of students are under undue stress of not performing well and taking the blame on themselves where their LD is to be diagnosed for the lack of performance. Self-referral and/or academic failure in the first year of medical college has been the most common means of identifying students who might have learning disabilities ( Rosebraugh, 2000).

### Attitudes and Disclosures

At one end of the spectrum, lies a student who is unaware of his LD and is facing difficult time in dealing with his studies while on the other end, another student is facing the same difficulty despite of knowing his LD due to the fear of disclosure and related stigma. It is extremely challenging for students to reveal their disabilities. “Fear of disclosure is real and prevalent” stated by Herzer (2017) in an article. The stigma associated with the word “disability” bars students from disclosing it as disclosure comes with risks and leads to negative consequences for the students ( Byron *et al.*, 2005).Another risk highlighted by the study exhibits that individuals with learning disabilities are alleged to have lower capabilities and competency levels ( Cole and Cawthon, 2015). Meanwhile, the true progress and accommodation requires the students to completely disclose the disability. Dr. Herzer emphasizes the significance of disability disclosure so that at one end, medical students are accommodated and facilitated by their medical schools and their rights are protected according to the law and at the other end, the attitudes regarding the word disability are corrected ( Byron *et al.*, 2005).

Cole and Cawthon (2007) conducted a mixed method study including quantitative questionnaires and a semi-structured interview with students having learning disabilities that revealed a significant difference in those who disclosed their disability in contrast to the non-disclosure group. The specific themes identified in the qualitative analysis of this study with the non-disclosure group were poor knowledge of accommodations, over-whelmingly negative view of their disability, faulty self-awareness that they did not need the accommodations and an apprehensive attitude to avoid negative comments/remarks from peers through non-disclosure ( Cole and Cawthon, 2015). In contrast, the students in disclosure group had a more positive view of their disability, their experience with the faculty and the demeanor of the professor ( Cole and Cawthon, 2015).

### Changing the attitudes about learning disabilities in medical students

In a study carried out in a medical school, students were asked to write two words that they associated the word “disability” with and not surprisingly, the words had a pessimistic connotation to it ( Byron *et al.*, 2005). After a 4- day course, the same group of students showed quite a remarkable difference in using words such as ‘empowerment’ and ‘independent’ for the same disability. Hall & Hollins (1996) conducted another study aimed to change the medical students’ view of disability by doing a workshop having people with Down’s syndrome in the role of teachers and leaders. The workshop improved the agreement on positive statements regarding the disability as compared to the negative statements.

Health professionals in general are considered to be insensitive and patronizing towards people with disabilities. This attitude is detrimental to those medical students who are afraid of disclosing their LD. These two studies show that medical students need to be empathetic towards adults with learning disabilities to be able to see the “person and not the disability.” Only then, the medical students having learning disabilities will be able to disclose it completely and will be viewed as valuable members of the community and applauded for their strength for coping with the stresses of the medical education despite their learning difficulty.

### Instructional and Assessment Approaches

Two of the problems identified in the instruction of SLD students is that the group is heterogenous with some having problems with reading, others with writing and so on and secondly that the educators lack knowledge about this condition ( Walters and Croen, 1993). The establishment of a Cognitive Skills Program, to identify academic difficulties faced by students, can play a significant role in identifying and providing students with appropriate accommodations to continue further learning ( Walters and Croen, 1993).

The best assessment approach has been that of multiple choice questions (MCQs) which caters for students with learning difficulties. According to a study, MCQs do not discriminate amongst students with LD, gender, or ethnicity ( Ricketts, Brice and Coombes, 2010). Students with writing disability also benefit from such assessment according to the view of educational psychologists and researchers. The Charter of Rights and Freedom (1982) and the Ontario Human Rights Code provide protection to people with disabilities. According to this, students with LD can have several reasonable accommodations made so that they have equal opportunity to perform well. Accommodations include provision of extra time, separate room to avoid distractions, color coded tests and use of assistive technology ( Ricketts, Brice and Coombes, 2010). One of the criticism to extra use of time is that any student will perform well under extra time, but research has shown that it has no added benefit to students without LD whereas substantial improvement is seen in those with LD ( Walters and Croen, 1993).

### Teaching medical students about learning disability and their assessment

According to Piachaud (2002), the core curriculum for medical students to learn about learning disability should be modelled around comprehensive knowledge, sufficient skills, and a positive attitude (p.336). The knowledge component can be covered in seminar or lecture format using video footage as an additional tool to sensitize students. Attendance of clinic with specialist learning disabilities service can also be a great learning experience ( Piachaud, 2002, p.338). For development of adequate skills, use of simulated patients and visits to clinics and services to assess and clerk the patients under supervision provides direct opportunity ( Piachaud, 2002, p.337). To foster a positive attitude, the students should have sufficient exposure to people with disabilities so that they learn to appreciate their strengths rather than their weaknesses. Pairing students with disabled people to have a real conversation, interaction with families having disabled children and attending workshops run by disabled people can help to dispel any negativity associated with disability. ( Piachaud, 2002, p.337). The assessments for this module can be done through simulated patients which can test their skills and attitudes and through MCQs which can assess the depth and breath of knowledge. ( Piachaud, 2002, p.340)

## Discussion

Learning disability becomes very challenging for the student and the problem doesn’t end when it is identified because even after identification, learning disability carries a stigma with it. The fear of being stigmatized and being discouraged further worsens their learning as proper accommodations cannot be made in time. Multiple studies show that learning disability should not be made a problem and every effort should be done to make a student a self-autonomous learner again. Provisions such as extra time, bold letters, color filter charts, separate room for examination etc. increase the test results of the students substantially. The principle of inclusive education dictates that we accommodate all types of learners in our classrooms so that the classroom is depiction of the real society where all kinds of people exist, and we cherish each other’s strengths and improve on each other’s weaknesses. Medical professionals are required to deal with people with disabilities and the curriculum places strong emphasis on how to deal with them as a person without patronizing them. Initially students did associate disability with negativity and sympathy but after some intervention, they did have a positive impact. It shows that regular courses and workshops should be held along with meeting people with disabilities so that students can carry a more empathetic view and are more sensitive to the issue.

I teach in a medical college in Pakistan and it is embarrassing and shocking for me to admit that most of the faculty members or the students of my college are unaware of the term “learning disability”. The only disability that is understood is the physical disability, which is looked upon as a defect or a deficiency. The concept of inclusive education is unknown. The attitudes of the faculty for the struggling student is simply that the “student is not trying hard enough” or the “student is lazy” ( Walters and Croen, 1993). The students themselves are unaware of their disability and struggle visibly more than their “able” counterparts.

One of the reasons for the discriminatory attitude of students and faculty towards disability is that there is no proper course or workshop that is conducted for this purpose. The students are not sensitized to the demands, strengths or living of the people with disabilities as they have had no exposure during their medical education. This is a major drawback, as future physicians in Pakistan, because some of the areas are conflict ridden zones and majority of the emergencies and general practice in those areas encounter people with disabilities. In context of learning disabilities, the empathy is even less as there is no physical disability to be seen. Students in this regard will benefit highly from courses and awareness workshops that inform them of prevalence of such conditions so that the magnitude of the problem is understood. According to the Government of Pakistan, there is a 2% reservation for the physically disabled people in governmental educational institutes and government jobs but ironically, people with learning disabilities are not included.

Another reason for not approaching the university for help is that there is no department or office looking out specially for such cases. Even if the student develops the courage to come forward with his problem, there is no relevant department or authority to take the case further. Such students can only be helped on an individual basis by the faculty if adequate resources allow. There is a need for development of special focal persons or relevant authority in every medical school so that such struggling students can be identified and focused upon. Special budget has to be allocated for the provision of accommodation to such students if identified early. The identification of such a disorder requires appropriate personnel to diagnose such condition which are currently not available in the medical schools. The concept of educational supervisor is unheard of and anyone who is going through a difficult academic time has no relevant authority to ask help from. Such is the deplorable case of medical education in Pakistan in terms of identifying learning disabilities and catering for them.

## Conclusion

Medical students with learning disabilities are gifted individuals who have come through this far without having an insight into their problem. Keen interest by the faculty and creating a positive atmosphere where students can come and discuss their academic problems is strongly required to diagnose this disability. Discrepancy between the student’s result and their understanding of the subject matter must be taken as a warning sign to probe further into the problem. Multiple choice questions (MCQs) prove to be the method of choice in assessment of people with learning disability as it does not discriminate between students with and without LD.

## Take Home Messages

Multiple choice questions (MCQs) prove to be the method of choice in assessment of people with learning disability as it does not discriminate between students with and without LD.

## Notes On Contributors

Dr. Arslaan Javaeed is an assistant professor of Pathology, in Poonch medical college, Rawalakot, and a student of Masters in Health Profession Education, University of Ottawa, Canada.

## References

[ref1] Ali K. Zahra D. Coelho C. Jones G. (2017) Academic performance of undergraduate dental students with learning disabilities. British Dental Journal. 222(3), pp.205–208. 10.1038/sj.bdj.2017.125 28184079

[ref2] Byron M. Cockshott Z. Brownett H. and Ramkalawan T. (2005) What does “disability” mean for medical students? An exploration of the words medical students associate with the term “disability”. Medical Education. 39(2), pp.176–183. 10.1111/j.1365-2929.2004.02062.x 15679685

[ref3] Cole E. V. and Cawthon S. W. (2015) Self-disclosure decisions of university students with learning disabilities. Journal of Postsecondary Education and Disability. 28(2), pp.163–179. https://files.eric.ed.gov/fulltext/EJ1074663.pdf

[ref4] Piachaud J. (2002) Teaching learning disability to undergraduate medical students. Advances in Psychiatric Treatment. 8(5), pp.334–341. 10.1192/apt.8.5.334

[ref5] Ricketts C. Brice J. and Coombes L. (2010) Are multiple choice tests fair to medical students with specific learning disabilities? Advances in Health Sciences Education. 15(2), pp.265–275. 10.1007/s10459-009-9197-8 19763855

[ref6] Rosebraugh C. J. (2000) Learning disabilities and medical schools. Medical Education. 34, pp.994–1000. https://onlinelibrary.wiley.com/doi/abs/10.1111/j.1365-2923.2000.00689.x 11123562 10.1046/j.1365-2923.2000.00689.x

[ref7] Walters J. A. and Croen L. G. (1993) An Approach to Meeting the Needs of Medical Students With Learning Disabilities. Teaching and Learning in Medicine. 5(1), pp.29–35. 10.1080/10401339309539584

