# Supplementary material for: Learning Disabilities and Medical Students
Source: MedEdPublish (2016). 2018 Jul 11;7:142. [Version 1] doi: 10.15694/mep.2018.0000142.1 (PMC10701842; doi:10.15694/mep.2018.0000142.1)
Supplement: Peer review history

# Reviewer report: Reviewer response for version 1

**DOI:** 10.21956/mep.19611.r29149

**Published:** 2018-07-16

**Authors:** Mohamed Al-Eraky

**Affiliations:** 1 University of Dammam

This review has been migrated. The reviewer awarded 4 stars out of 5 I enjoyed reading this nice paper that alerts educators to attend to the growing prevalence of learning disabilities, which reached 6% in higher education, as cited in some studies. The article can be supplemented to take the shape of one of two manuscripts: (1) A full-scale study, in case if the author chooses to conduct and report an intervention to manage learning disabilities in his institution, (2) A review article, where he will need to attend to the points raised by my colleague, Julie Williamson in her comments, e.g. selection criteria of papers and adding a global perspective to the Discussion. I would further suggest to support the personal views, observations and assumptions, such as: ‘the concept of inclusive education is unknown’ (in Pakistan) with evidence to support authors’ argument and engage the reader. In the Discussion section, the concepts and ‘facts’ presented in the 3rd and 4th paragraphs need to be properly cited. Nice work! This can be a template for a very nice article. Good luck.

Reviewer Expertise:

NA

No decision status is available

# Reviewer report: Reviewer response for version 1

**DOI:** 10.21956/mep.19611.r29151

**Published:** 2018-07-15

**Authors:** Sateesh Babu Arja

**Affiliations:** 1 Avalon University School of Medicine

This review has been migrated. The reviewer awarded 4 stars out of 5 I thoroughly enjoyed reading this paper. The writing style is simple and well written. The author set up the background and introduction very well. It is sad to see that learning disability is one of the key areas in the medical education which is often neglected. In my experience, students with learning disabilities have come forwarded and asked for the additional help. They have to go through the documentation and additional procedures. I have seen students with attention deficit disorder and attention deficit hyperactive disorder asking for additional time and separate rooms during exam time. But I have never come across the students with dyscalculia or dyslexia in the medical school. The author also reviewed the solutions for teaching methods and assessment methods to help the students with disabilities. This paper is useful to the academic faculty members, academic administrators and students affairs office.

Reviewer Expertise:

NA

No decision status is available

# Reviewer report: Reviewer response for version 1

**DOI:** 10.21956/mep.19611.r29148

**Published:** 2018-07-14

**Authors:** sathyanarayanan varadarajan

**Affiliations:** 1 SRM Medical College, SRM University

This review has been migrated. The reviewer awarded 4 stars out of 5 In this interesting article, the author explores a relatively neglected area in medical education, the Learning Disabilities and Medical Students. He has highlighted different learning disabilities and their effects on medical students and suggests the best assessment strategy for such students. He has done this by searching the medical education literature for articles related to learning disabilities and how they affect medical students. He discusses in detail on the Attitudes and Disclosures of students with learning disabilities, How to change the attitudes about learning disabilities in medical students, Teaching medical students about learning disability, their assessment and Instructional and Assessment approaches for them. He presents some important facts like “ Half of the individuals with Learning Disabilities are not identified before they start their college” and “Self-referral and/or academic failure in the first year of medical college has been the most common means of identifying students who might have learning disabilities” etc. He strongly suggests the best assessment approach for students with learning difficulties is multiple choice questions (MCQs). This article will be particularly helpful to the medical educators who are involved in Curriculum Planning and development, faculty involved in teaching and assessment of medical students, Student Mentors and Student Counselors...

Reviewer Expertise:

NA

No decision status is available

# Reviewer report: Reviewer response for version 1

**DOI:** 10.21956/mep.19611.r29152

**Published:** 2018-07-12

**Authors:** P Ravi Shankar

**Affiliations:** 1 American International Medical University

This review has been migrated. The reviewer awarded 4 stars out of 5 I read with great interest the article on learning disabilities. The author provides an overview of learning disabilities. I agree with the author that learning disabilities are not commonly recognized in developing nations and even, if they are, support mechanism for these students are lacking. When I was working in Nepal I was aware of learning disabilities but did not come across students diagnosed with the same. In developing countries there still continues to be a stigma associated with disability. In the Caribbean, learning disabilities are diagnosed among students in offshore medical schools and the most common accommodation provided is extra time to complete the exam. I would be interested in knowing why the author considers multiple choice questions to be the best method of assessment for these individuals? It takes courage for medical students to acknowledge learning disabilities and seek support in developing countries. Developing countries have a certain percentage of seats reserved for physically disabled candidates but not for those with learning disabilities. Most medical schools in the developing world are not disabled friendly. When I was doing my undergraduate medical education in India in the late 80s I did not come across students identified with learning disabilities. Language corrections may be required in a few places in the manuscript. The article will be of interest to a broad spectrum of medical educators.

Reviewer Expertise:

NA

No decision status is available

# Reviewer report: Reviewer response for version 1

**DOI:** 10.21956/mep.19611.r29150

**Published:** 2018-07-11

**Authors:** Julie Hunt

**Affiliations:** 1 Lincoln Memorial University

This review has been migrated. The reviewer awarded 3 stars out of 5 This article brings together and summarizes previous work on learning disabilities in medical students. The author provides an excellent description of the scope and impact of learning disabilities experienced by medical students. As it is a review paper, I would have liked to have heard more about how studies were searched and screened for inclusion. The discussion section talks of the situation in Pakistan in particular, which doesn't seem to fit with the global nature of the rest of the paper. Adjustments to the title and headings may clarify that the paper is in fact two parts: an exploration of learning disabilities in medical students, and a commentary on the situation in Pakistan. It troubled me to hear medical educators worldwide and in Pakistan being painted as insensitive to learning disabilities. I hope that better understanding this topic will help educators to recognize that accommodations are necessary for fairness in education.

Reviewer Expertise:

NA

No decision status is available
